# Systemic hematogenous dissemination of mouse oral candidiasis is induced by oral mucositis

**DOI:** 10.1007/s10266-018-0366-1

**Published:** 2018-05-24

**Authors:** Hiroki Katagiri, Kayoko Fukui, Kenjirou Nakamura, Akira Tanaka

**Affiliations:** 1 Course of Clinical Science, Field of Oral and Maxillofacial Surgery and Systemic Medicine, Oral and Maxillofacial Surgery, The Nippon Dental University Graduate School of Life Dentistry at Niigata, 1-8 Hamaura-cho, Chuo-ku, Niigata, 951-8580 Japan; 2 Department of Pharmacology, The Nippon Dental University School of Life Dentistry at Niigata, Niigata, Japan; 3 Department of Oral and Maxillofacial Surgery, The Nippon Dental University School of Life Dentistry at Niigata, Niigata, Japan; 4 Division of Cell Regeneration and Transplantation, Advanced Research Center, The Nippon Dental University School of Life Dentistry at Niigata, Niigata, Japan

**Keywords:** Fungemia, Mouse oral candidiasis, Oral mucositis, Oral care, *Candida albicans*

## Abstract

The causes of fungemia include immunosuppression and neutropenia stemming from diverse factors as well as the placement of central venous catheters. However, the relationship between fungemia and the oral cavity has not been substantiated. In this study, we explored the pathological conditions of *Candida albicans—*derived oral candidiasis in a mouse model, which always develops oral mucositis as a complication. In oral candidiasis, the hyphae of *C. albicans* are believed to primarily invade the stratum granulosum, but not the subepithelium, of the mucous membrane. We provide histological evidence that in concomitant oral mucositis, the hyphae infiltrate the subepithelium and blood vessels. Blood cultures and tissue samples revealed the onset of fungemia only in the mucositis-induced groups. Positive numbers of colony-forming units were found in groups A (chemotherapy), B (chemotherapy + mucositis) and C (mucositis), but were highest in group B. Some organs revealed positive CFU in groups B and C. The presence of fungal DNA in blood plasma and tissue was confirmed by PCR. The fungal DNA frequency was significantly higher in the mucositis group when compared with the non-mucositis group. The results suggest that fungi first invade the subepithelium and then the blood vessels, from which they disseminate throughout the body, and that oral mucositis is an important risk factor for fungemia. This study clearly demonstrates the relationship between oral mucositis, fungemia, and the potential systemic fungal dissemination, which has not been previously proven. Our findings highlight the importance of oral care for patients at risk of fungemia.

## Introduction

Superficial mycoses such as oral candidiasis can progress to deep mycoses such as fungemia, which are sometimes fatal. As these conditions can affect the success rate of cancer treatment, controlling their onset is vital. In oral candidiasis, *Candida* hyphae are generally believed to penetrate only the superficial layers of the mucous membrane. We have been exploring the pathogenicity of *Candida albicans* and *Candida dubliniensis* [1], and their influence on the mucous epithelium, by experimentally developing a mouse model of oral candidiasis. So far, hyphal infiltration of the subepithelium and subsequent deep mycosis has not been observed. However, oral mucositis is an adverse event that occurs in approximately 40% of patients receiving chemotherapy with anticancer medication, and in almost 100% of patients receiving radiotherapy to the oral area [2, 3]. Radiation-derived oral mucositis is reportedly aggravated by candidiasis [4]. Nevertheless, details such as the correlation between these diseases and how mucositis progresses to deep mycosis that disseminates throughout the body remain unknown.

In the present study, we experimentally developed a new mouse model of oral candidiasis for reproducing human combination chemotherapy by intraperitoneal administration of cisplatin (CDDP) and 5-fluorouracil (5-FU). Both are standard chemotherapy medications for head and neck cancer. To examine the effects of CDDP and 5-FU on oral candidiasis and the body, we experimentally ensured that the mice would contract oral mucositis as a complication of the treatments.

## Materials and methods

The study was approved by the Animal Experimentation Ethics Committee of The Nippon Dental University School of Life Dentistry at Niigata (Approval No. 95).

### Animals

Experimental animals were 8-week-old male ICR mice (CLEA Japan, Tokyo, Japan). The mice were housed at 24 °C and given free access to food and water. For infection control, 0.83 g/L tetracycline hydrochloride (Waco Pure Chemical Industries, Tokyo, Japan) was added to the water. To prevent microbial contamination, all experimental apparatus, including litter and rearing containers, were sterilized in advance. Among the *C. albicans* strains held by the Advanced Research Center at the Nippon Dental University, Niigata, we selected the IFM40009 (ATCC 48130) strain, which yielded pathological factors of the fungus (high protease and phospholipase activity) [1]. The experimental mice were divided into four groups: group A (chemotherapy alone), group B (chemotherapy + mucositis), group C (mucositis alone), and a negative control group without microbial/drug administration and mucositis induction (*n* = 10 per group). Each group was orally inoculated with *C. albicans* (5.0 × 10^6^ cells/25 µL) at 2, 3, and 5 days after starting chemotherapy [5]. Separately from each experimental group, the white blood cell (WBC) count of non-inoculated mice was measured before and after chemotherapy using an automatic blood cell counting device (KX-21N; Sysmex Corp., Hyogo, Japan). This test ascertains changes in the WBC count induced by chemotherapy alone (*n* = 7). Starting from 1 day before chemotherapy (day 0), the body weight of each mouse was recorded at the same time every day.

### Experimental schedule (Fig. 1)

**Fig. 1:**
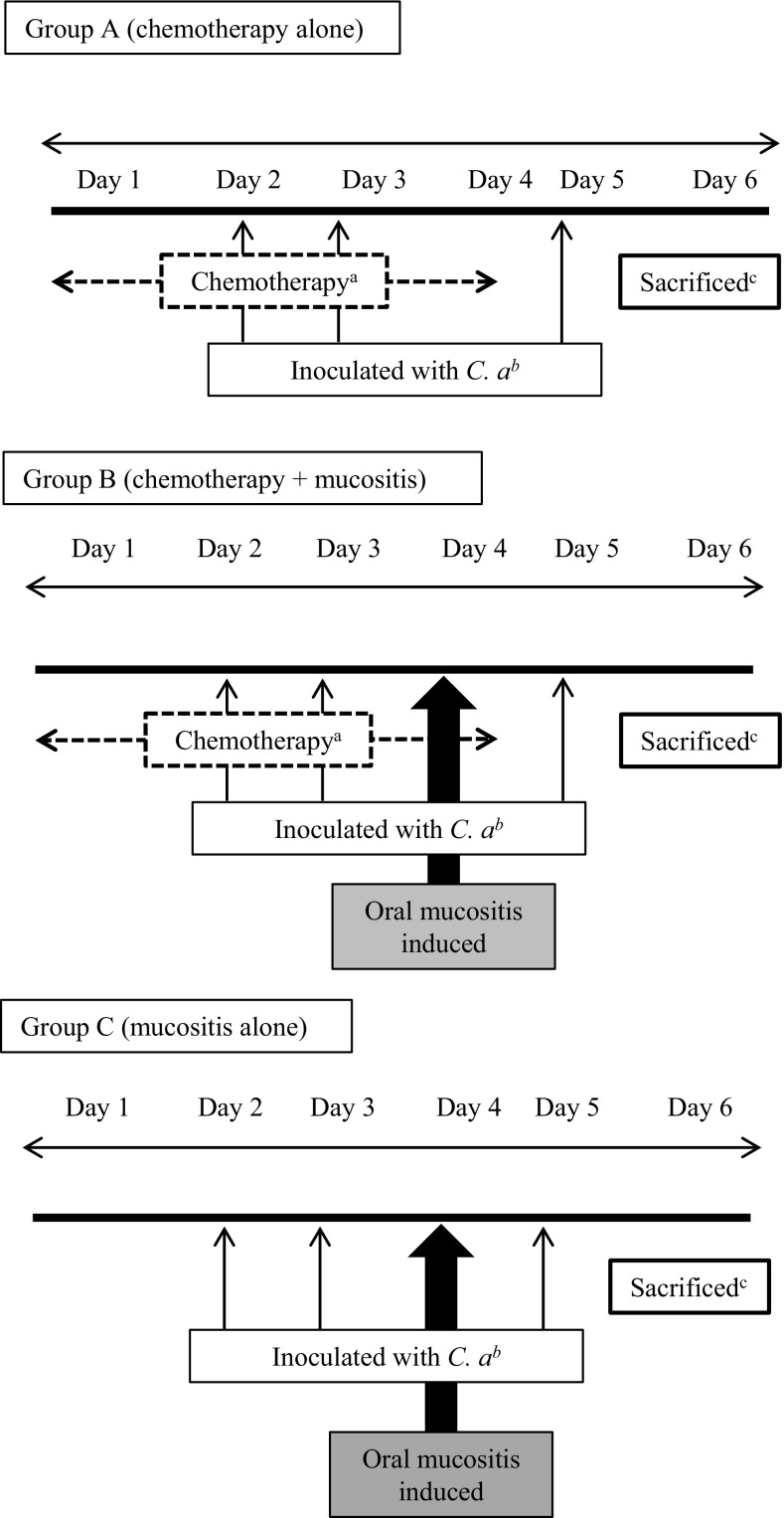
Experimental schedule. **a** Chemotherapy groups were intraperitoneally administered 7 mg/kg CDDP on day 1, and 10 mg/kg 5-FU on days 1, 2, 3, and 4. **b** Each group was then orally inoculated with *C. albicans* (5.0 × 10^6^ cells/25 µL) 2, 3 and 5 days after starting chemotherapy, according to the method by Takakura et al. [5]. **c** On day 6, the mice were sacrificed and their organs (tongue, liver, and kidneys) and blood samples were collected aseptically from the cardiac apex [8]

**Fig. 2:**
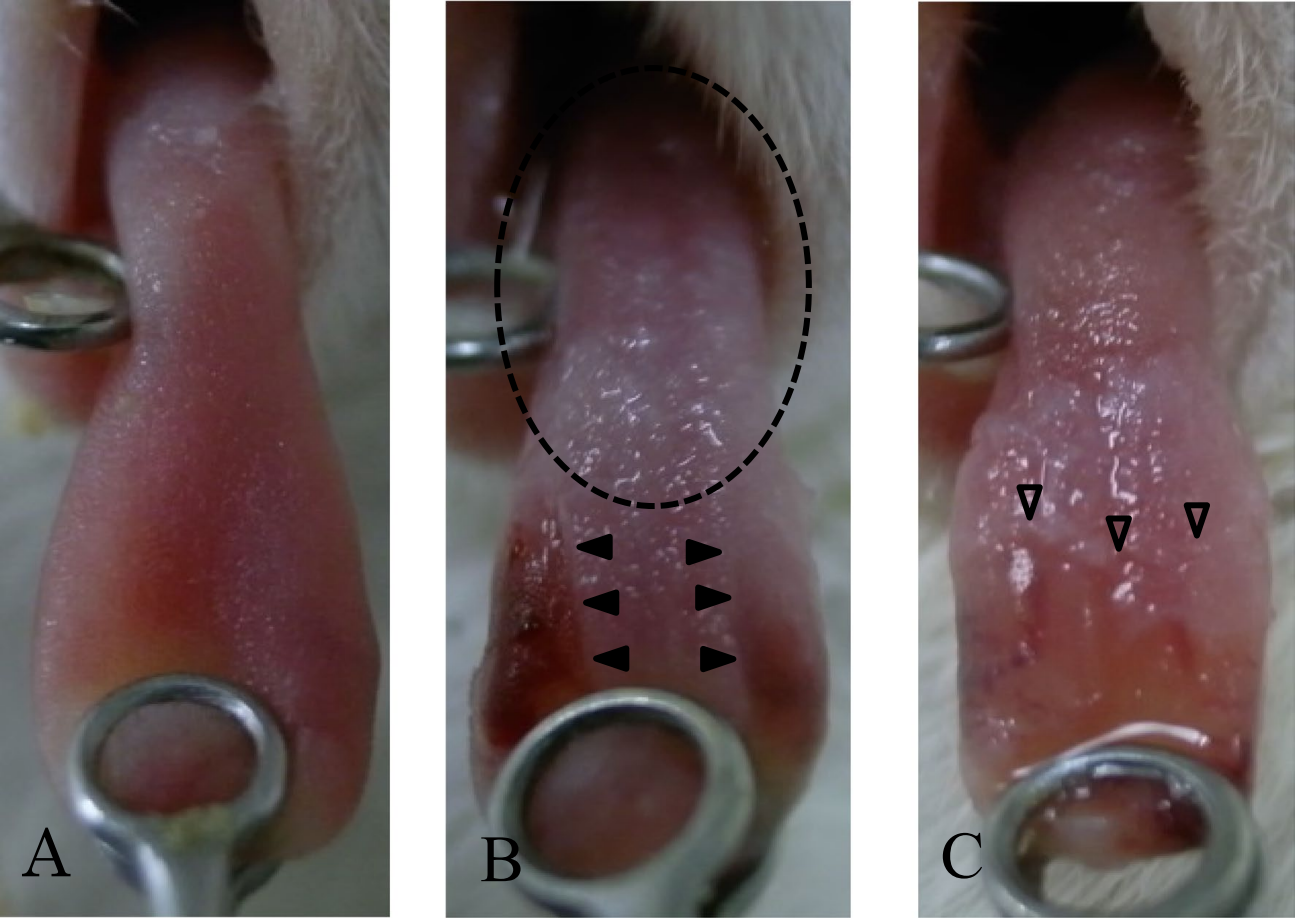
**a** Day 0: before the experiment. **b** Day 4: mucositis was induced on both sides of the lingual border (arrowhead). The presence of oral candidiasis was indicated by white patches on the tongue (circle shown by a broken line). **c** Day 6: expansion of mucositis (white arrowhead)

Chemotherapy groups were intraperitoneally administered 7 mg/kg CDDP (Nichi-Iko Pharmaceutical, Toyama, Japan) on day 1, and 10 mg/kg 5-FU (Kyowa Hakko Kirin, Co., Ltd., Tokyo, Japan) on days 1, 2, 3, and 4. Mucositis was induced as described by Yun-Sung et al. [6] and Fujisawa et al. [7]. First, oral candidiasis in the mice (day 4 of chemotherapy) was macroscopically ensured. Next, the mice were anesthetized with general anesthetic (concomitant pentobarbital and 2% xylazine hydrochloride), and surface anesthetic (xylocaine jelly) was applied to the tongue of each mouse. Finally, cotton swabs infused with 25 µL 50% acetic acid were spotted on each side of the lingual border for 30 s to 1 min. To remove any remaining drugs, the area was thoroughly washed with cotton swabs infused with sterile saline (Fig. 2). Once mucositis was induced, the mice were given free access to food that was crushed into muddy pellets with water. On day 6 of chemotherapy (5 days after inoculation), the mice were sacrificed by overdosing with general anesthetic (pentobarbital), and their organs (tongue, liver, and kidneys) and blood samples were collected aseptically from the cardiac apex [8]. After centrifugation (3000 rpm × 5 min), the plasma portion of the collected blood was sampled for polymerase chain reaction (PCR).

### Culture and histological studies

After washing the organs with sterile saline and weighing them, half the sagittal section of the tongue, the right half of the kidney, and part of the liver near the bile duct were homogenized with sterile saline, yielding a 1-mL solution in a sterile micro-tube (BioMasher II; Assist, Tokyo, Japan). Subsequently, 100 µL of the solution was spread onto a Sabouraud agar plate (Nissui Pharmaceutical, Tokyo, Japan) and incubated at 30 °C for 48 h. After incubation, colony-forming units (CFU) were counted. The other half of the organ sample was immersed and fixed in 4% neutral buffered formalin solution for 48 h. After embedding in paraffin using the standard method, specimens were stained with hematoxylin–eosin (H–E) and periodic acid–Schiff (PAS) stains. The tongue specimens were additionally subjected to immunohistochemical study (Von Willebrand factor staining).

### Blood culture

Blood samples were aseptically collected upon death of the mice and incubated in peptone yeast extract glucose liquid medium at 30 °C for 72 h. Samples were then spread on a Sabouraud agar plate (Nissui Pharmaceutical, Co., Ltd., Tokyo, Japan) and incubated at 30 °C for 48 h. Following culture (5 days in total), fungal growth was checked by observing colonies on the plate.

### Molecular analysis

To check whether the strain in the blood, liver, and kidney specimens that tested positive for CFU was identical to the inoculated strain, CFU strains were identified by multiplex PCR [9] (Table 1a). Similarly, the presence of *C. albicans*–specific DNA in blood plasma and tissue was detected by nested PCR. DNA was purified using DNA purification kits (Norgen Biotek Corp., Thorold, Canada) [10] (Table 1b).

**Table 1:** Sequence of PCR primers

Identification of *Candida* spp.	Sequence (5′ to 3′)	Gene name	GenBank no.	Amplified size (bp)
a. Oligonucleotides used in multiplex PCR [9]				
*C. albicans*	albF GCTCGCATATACCTGTCATTG	*SAP5*	AF043548	615
	albR CGAGCTTGCCATTTGAATG			
*C. dubliniensis*	dubF GGCTCATCTATTTTAGCTAC	*HWP1*	AJ632273	416
	dubR CCTGGAGCCGATTCTGTAGT			
*C. glabrata*	glaF ATGTCCACTGAAAACACTTCTTTG	*ERG11*	L40389	1006
	glaR CTGGTCCTTTCAGCCAAATGC			
*C. guilliermondii*	guiF GATCCACAGGAACATTATCGATG	*XYL1*	DQ297454	512
	guiR CATGACTAAAATGGACCAC			
*C. krusei*	kruF ACCTTGATCCAGTTGCTTAC	*ABC1*	DQ903906	1298
	kruR CTCGTGGTAGTCCTGGTTC			
*C. parapsilosis*	parF GCTGTTGGATTGTGTCATTCTG	*rCR*	DQ295067	833
	parR GGCAATTCCTTCAATTTGGCAC			
*C. tropicalis*	troF GGACGGGGGTATGTTTCAATTAAATC	*ACT1*	AJ237918	327
	troR CCGATTACAGATAAGTAATTTCC			

	Sequence (5′ to 3′)	Fragment size (bp)		
b. Primers used in nested PCR for *C. albicans* [10]				
Outer primer				
Sense	TCCGTAGGTGAACCTGCGG	750		
Antisense	TCCTCCGCTTATTGATATGC			
Inner primer				
Sense	AACTTGCTTTGGCGGTGGGC	386		
Antisense	TGGACGTTACCGCCGCAAGC			

### Statistical analysis

WBC counts were analyzed using Student’s *t* test. Body weights of mice were analyzed using two-way analysis of variance, tongue CFU were analyzed using Steel–Dwass multiple comparisons, and organ CFU were analyzed using Steel–Dwass multiple comparisons between each group, and Wilcoxon signed-rank tests between organs. Blood cultures and the results of nested PCR were analyzed using the Benjamini–Hochberg method for multiple comparisons. Statistical analyses were performed using the statistics add-in for BellCurve for Excell (Social Survey Research Information Co., Ltd, Tokyo, Japan), and R ver. 3.3.2 software.

## Results

### Changes in WBC count and body weight

WBC counts were 2142 ± 292/µL (mean ± SD) in the chemotherapy (non-inoculated) group and 3542 ± 629/µL in the non-chemotherapy (healthy) group. Chemotherapy reduced the WBC count by approximately 39.5%. The difference between the chemotherapy group and the non-chemotherapy group was statistically significant (*P* < 0.01). In each group, weight loss was gradually observed from day 3 when compared with the control group, and a significant difference was observed only in group B. At day 4, significant differences were observed between groups A, B, and C and the control group. Group B showed the most prominent weight loss among the groups.

### Macroscopic findings

In all groups, typical lesions consisting of white patches on the tongue were gradually observed, and remained throughout the experiment. In addition, the lesions increased when oral mucositis was induced (Fig. 2, groups B and C).

### Histopathological findings

**Fig. 3:**
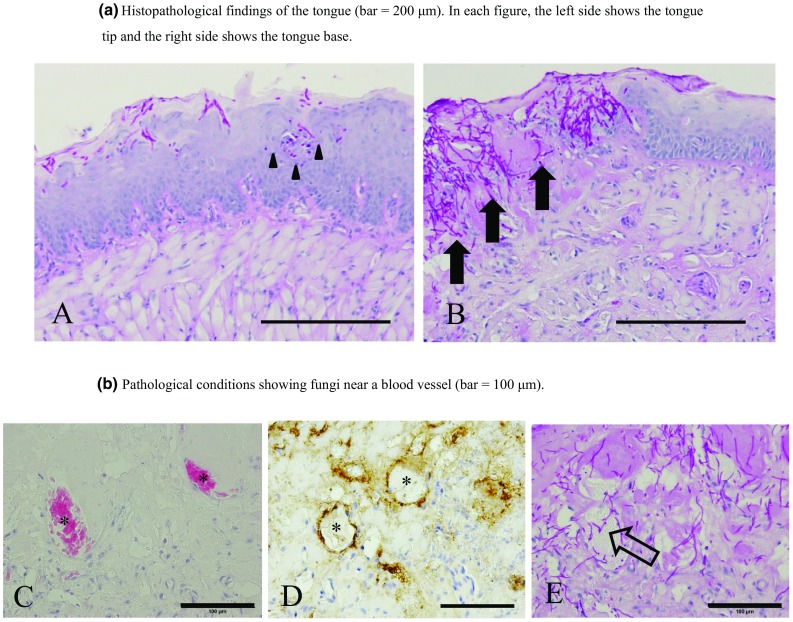
**a, b** PAS staining (× 200). **a** Group A: the *Candida* hyphae have not progressed into the subepithelium (arrowhead). **b** Group B: a large amount of *Candida* hyphae invades the subepithelium of the tongue from the defective part of the basal layer (arrow). **c, d** H–E staining, Von Willibrand factor staining (× 400). Identification of blood vessels in the tissue; asterisk shows blood vessels. **e** *Candida* hyphae infiltrate the blood vessels. PAS staining (× 400) (white arrow)

Breakdown of the basal layer was identical in groups B and C. In the same area, PAS staining confirmed that numerous fungi were present on the boundary between the damaged and unaffected sections of the basal layer. Fungi had invaded the subepithelium (Fig. 3b). Immunohistochemical staining (Von Willebrand factor staining) and PAS staining revealed intravascular hyphae in the vascular area (Fig. 3c–e).

### Fungal counts in the tongue and organs

**Fig. 4:**
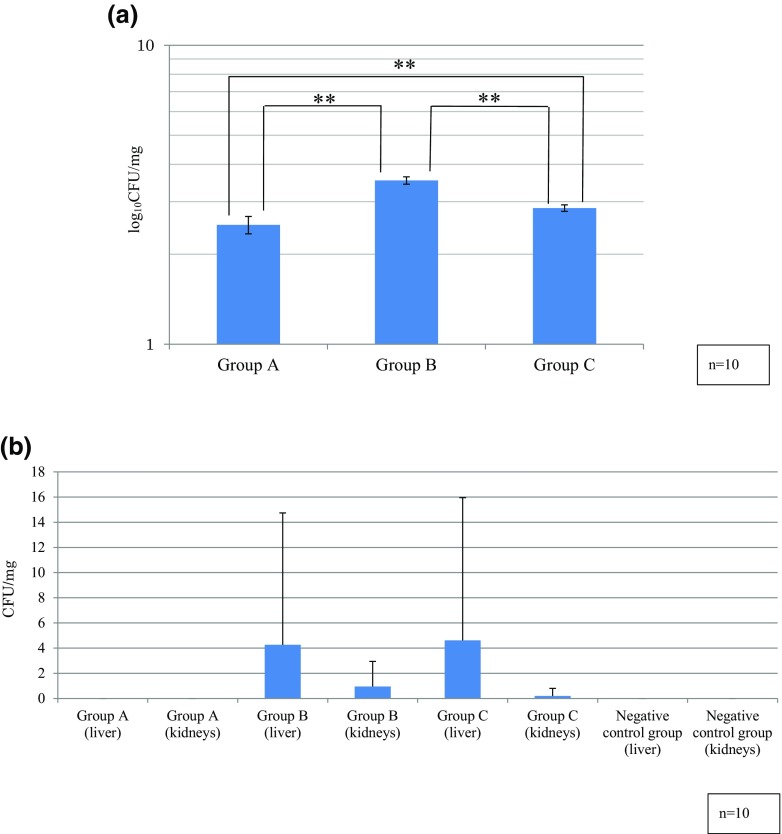
**a** Tongue log_10_ CFU/mg. The highest colony-forming unit (CFU) counts were obtained in group B. Between-group comparisons were statistically significant (**P < 0.01). **b** Organ CFU/mg. CFU counts were lower in the liver and kidney specimens when compared with tongue specimens. No statistically significant difference was observed

Fungal counts in the tongue were 2.51 ± 0.17 (mean ± SD) log_10_CFU/mg in group A, 3.53 ± 0.10 log_10_CFU/mg in group B, and 2.85 ± 0.07 log_10_CFU/mg in group C. Counts were higher in group B when compared with the other groups. The differences between all groups were statistically significant (Fig. 4a).

In the liver and kidneys, the mucositis-induced groups were CFU-positive, albeit at smaller numbers that were not statistically significant (Fig. 4b).

### Blood culture

Ratios of CFU-positive specimens in blood cultures were 0/10 (positive/total) in group A, 6/10 in group B, 3/10 in group C, and 0/10 in the negative control group. Fungemia occurred only in the mucositis-induced groups (groups B and C), and the incidence rate was higher in the group with concurrent chemotherapy (group B). Blood culture results were significantly different between groups A and B, and between group B and the negative control group.

### Multiplex and nested PCR

**Fig. 5:**
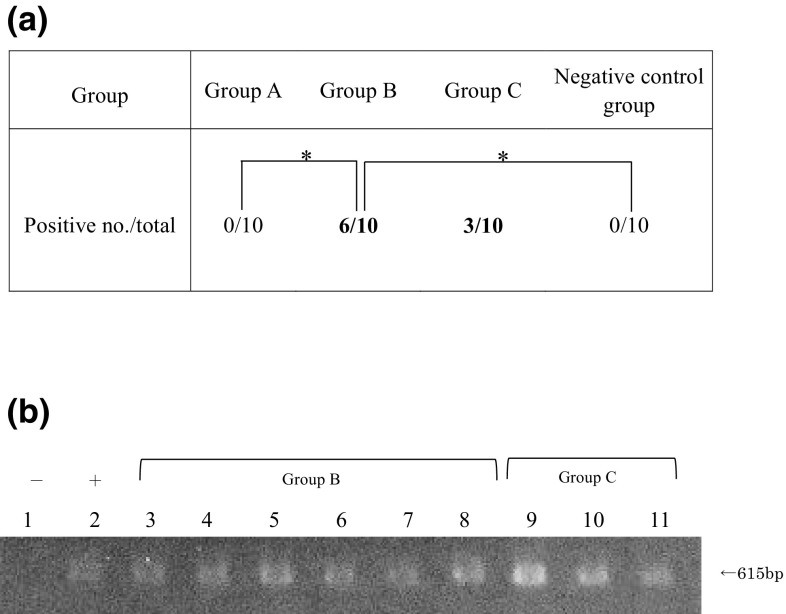
**a** Blood culture analysis. Only the mucositis-induced groups (groups B and C) yielded positive results in blood culture (*P < 0.05). **b** Positive results of multiplex PCR for blood cultures. The 615 bp band specifically identifies *Candida albicans* among *Candida* spp. Lane 1: negative control (buffer), lane 2: positive control (*C. albicans*), lanes 3–8: group B, and lanes 9–11: group C

Strains in blood serum, liver, and kidney specimens that tested positive for CFU in blood cultures were identified by multiplex PCR. All specimens harbored the inoculated *C. albicans* strain IFM40009 (Fig. 5b).

**Fig. 6:**
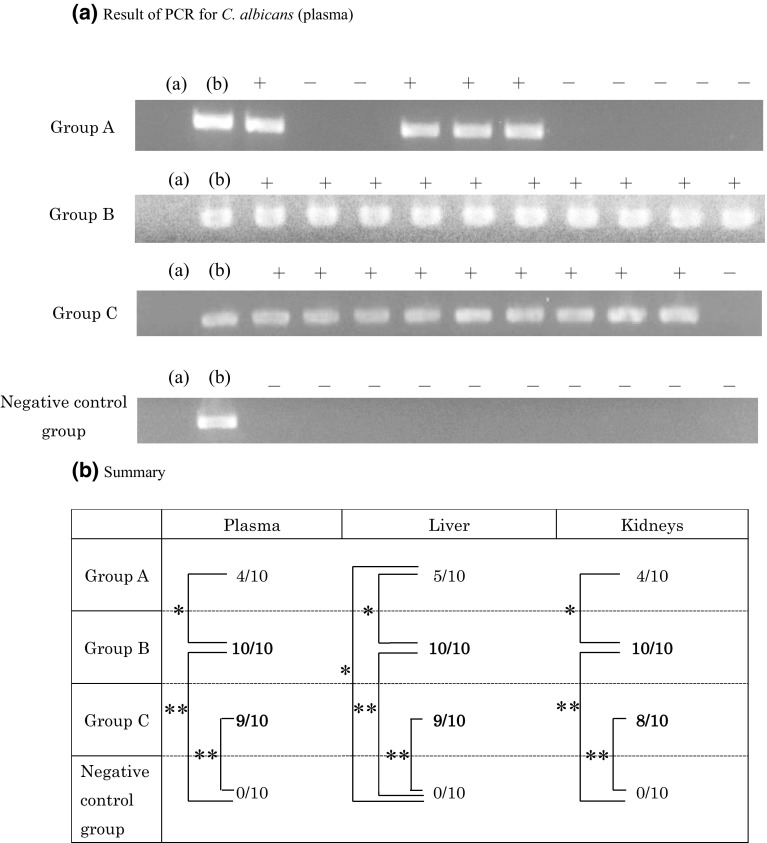
**a** Lane a: negative control (buffer), lane b: positive control (*C. albicans*), lane to the right indicates positive or negative. **b** Occurrence (relative to all samples) was elevated in the mucositis-induced groups and was highest in group B. A similar trend was observed in liver and kidney specimens, but all specimens in the non-inoculated groups tested negative (*P < 0.05, **P < 0.01)

Although nested PCR revealed CFU-positive blood plasma specimens in all fungus-inoculated groups, the occurrence was more frequent in the mucositis-induced groups. The CFU-positive ratio was again higher in group B when compared with the other groups. A similar trend was observed in the liver and kidney specimens. All specimens in the non-fungus-inoculated groups tested negative. The ratios in blood plasma, liver, and kidney specimens were significantly different between groups A and B (Fig. 6a, b).

## Discussion

Antineoplastic chemotherapy using CDDP and 5-FU in humans is the standard treatment for oral cancer [11, 12]. However, this treatment does have adverse effects, such as immunosuppression because of leukopenia and mucositis, which tend to occur almost simultaneously [2, 3, 13]. Furthermore, there have been clinical reports of fungemia occurring with CDDP and 5-FU chemotherapy [14].

The general procedure to create the murine model of oral candidiasis is to use chlorpromazine to adhere candida in the oral cavity. However, the fungus does not exist in the mouse as part of its normal flora, so even if it does adhere, it will be eliminated by the natural and cellular acquired immunity [15]. As such, it does not adhere for long and invasion of the hyphae are only known to reach the granular layer of the epithelium [16, 17]. In the current study, we succeeded in developing a mouse model of oral candidiasis by administering the immunosuppressant antineoplastic drugs CDDP and 5-FU in addition to chlorpromazine. 5-FU has antifungal effects, and flucytosine, an antifungal drug, is one of its prodrugs. For this reason, there is concern over the impact of its antifungal effect when administering 5-FU as an anticancer drug to create murine oral candidiasis. The minimal inhibitory concentration (MIC50) of 5-FU on *C. albicans in vitro* is reported to be 100 µg/mL [18]; however, when 200 mg/kg 5-FU was administered intraperitoneally to mice, the concentration in the blood was only 0.39 µg/mL [19]. Therefore, we anticipated that the concentration of the drug we administered, expecting anticancer effects, would not impact the antifungal effect. In the current study, there were also oral candidiasis complications in mice in the chemotherapy-only group; therefore, we believe that this did not impact the experimental model.

*Candida* is not an indigenous oral microbe of the conventional laboratory mouse [17]. We hypothesized that normal mouse oral bacterial flora may prevent *C. albicans* colonization in the mouse oral cavity. Therefore, we used a relatively high dose of tetracycline hydrochloride (0.83 g/L) to eliminate the bacterial flora as soon as possible before the first inoculation of *C. albicans* in accordance with the method established by Takakura et al. [5].

We induced oral mucositis following the method by Sonis et al. [20] without physical stimulation, but with chemical stimulation using acetic acid to destroy the basal layer of the epithelium. We prepared the acetic acid solution at a 50% concentration based on the mucositis induction method by Yun et al. and Fujisawa et al. [6, 7]. With H–E staining, as we observed destruction only in the basal layer, we determined that we had succeeded in reproducing clinical oral mucositis.

Fungi are part of the normal flora of the human oral cavity, skin, and urinary and enteral tracts, and the carrier rate increases with age [21, 22]. Fungi are known to be pathogenic microorganisms causing septicemia and are reportedly observed in 15% of septicemia cases [23–26]. A delayed diagnosis can be lethal, and once a person is affected by fungemia, the fatality rate is high (40%). Other studies have reported that the rate is as high as 74% [27, 28]. The mortality rate is also reported to increase with delayed introduction of antifungal drug administration [29]. There are a variety of risk factors [30], and according to reports to date, placement of a central venous catheter is the most significant risk factor contributing to onset; however, there have been no studies that have elucidated the relationship with oral mucositis [31].

In the current study, our observations (fungal invasion into the subepithelium of the tongue and migration to the blood in a viable form, significant increases in the rate of fungemia onset, and discovery of viable fungus in certain organs) suggest that oral mucositis may be a pathway for fungi to enter the bloodstream. Using nested PCR, we found *C. albicans* DNA present in significant amounts in the blood and tissue in the presence of oral mucositis. It was also detected at significantly higher levels in the chemotherapy-only group when compared with the control group. Using PCR, DNA of dead microorganisms can also be detected; therefore, it is not known whether specimens that tested positive had viable fungus at the time of tissue collection. Specific DNA detection suggests that fungemia occurred, even if transitory, and that some fungi were in deep tissue. From this experiment, we confirmed significant differences in blood cultures, blood plasma, and hepatic and renal findings between the chemotherapy-only and chemotherapy and mucositis groups. This suggests the potential for complications when mucositis occurs during chemotherapy.

In the chemotherapy-only group, we did not observe the natural onset of mucositis; however, we observed fungemia in certain individuals. This suggests that the fungus may have entered either orally and proliferated after adhering; by direct entry via enteral tract mucositis, an adverse effect of anticancer drugs; or by the enteric–hepatic circulation; or it may have entered the blood by microbial translocation [32], among other possibilities, to migrate throughout the body.

Past studies have reported that pathogenic fungus and fungus detected from the oral cavity matched in 60% of clinical fungemia cases [33, 34]. In the current study, we found that oral mucositis was another important pathway for entry of fungus. Being a candida carrier is associated with poor oral hygiene; therefore, oral care is an important factor to control.

The study also suggests the importance of screening for carriers of fungus in addition to oral cavity management such as recontouring any sharp alveolar ridge and adjusting ill-fitting dental prostheses, which can induce decubitus ulcers of the oral mucosa, prior to starting chemotherapy.

## Conclusion

This study examined the relationship between oral candidiasis and hematogenous dissemination. As a result, In the presence of oral mucositis, fungi revealed the possibility of hematogenous dissemination. This suggests the importance of oral care not only for patients undergoing cancer treatment but for all patients at risk of fungemia.
